# Quantitative evaluation and modeling of two-dimensional neovascular network complexity: the surface fractal dimension

**DOI:** 10.1186/1471-2407-5-14

**Published:** 2005-02-08

**Authors:** Fabio Grizzi, Carlo Russo, Piergiuseppe Colombo, Barbara Franceschini, Eldo E Frezza, Everardo Cobos, Maurizio Chiriva-Internati

**Affiliations:** 1 Scientific Direction, Istituto Clinico Humanitas, Via Manzoni 56 – 20089 Rozzano, Milan, Italy; 2 "Michele Rodriguez" Foundation-Institute for Quantitative Measures in Medicine, Via Ludovico Di Breme 79 – 20100 Milan Italy; 3 Department of Pathology, Istituto Clinico Humanitas, Via Manzoni 56 – 20089 Rozzano, Milan, Italy; 4 Department of Surgery, Texas Tech University Health Science Center and the Southwest Cancer Treatment and Research Center, 79430 Lubbock, Texas, USA; 5 Department of Internal Medicine, Texas Tech University Health Science Center and the Southwest Cancer Treatment and Research Center, 79430 Lubbock, Texas, USA; 6 Department of Microbiology and Immunology, Texas Tech University Health Science Center and the Southwest Cancer Treatment and Research Center, 79430 Lubbock, Texas, USA

## Abstract

**Background:**

Modeling the complex development and growth of tumor angiogenesis using mathematics and biological data is a burgeoning area of cancer research. Architectural complexity is the main feature of every anatomical system, including organs, tissues, cells and sub-cellular entities. The vascular system is a complex network whose geometrical characteristics cannot be properly defined using the principles of Euclidean geometry, which is only capable of interpreting regular and smooth objects that are almost impossible to find in Nature. However, fractal geometry is a more powerful means of quantifying the spatial complexity of real objects.

**Methods:**

This paper introduces the *surface fractal dimension (D_*s*_) *as a numerical index of the two-dimensional (2-D) geometrical complexity of tumor vascular networks, and their behavior during computer-simulated changes in vessel density and distribution.

**Results:**

We show that *D*_*s *_significantly depends on the number of vessels and their pattern of distribution. This demonstrates that the quantitative evaluation of the 2-D geometrical complexity of tumor vascular systems can be useful not only to measure its complex architecture, but also to model its development and growth.

**Conclusions:**

Studying the fractal properties of neovascularity induces reflections upon the real significance of the complex *form *of branched anatomical structures, in an attempt to define more appropriate methods of describing them quantitatively. This knowledge can be used to predict the aggressiveness of malignant tumors and design compounds that can halt the process of angiogenesis and influence tumor growth.

## Background

The term "angiogenesis" defines the fundamental process of the development and growth of new blood vessels from the pre-existing vasculature, and is essential for reproduction, development and wound repair [1]. Under these conditions, it is highly regulated: *i.e. *"turned on" for brief periods of time (days) and then completely inhibited.

The cyclic nature of the microvascular bed in the corpus luteum provides a unique experimental model for examining the discrete physiological steps of angiogenesis in the life cycle of *endothelial cells *which, together with *pericytes *(supportive vascular smooth muscle cells), carry all of the genetic information necessary to form *tubes*, *branches *and entire *capillary networks*.

However, many human diseases (including solid tumors) are driven by persistently up-regulated angiogenesis [1]. In some non-malignant processes, such as pyogenic granuloma or keloid formation [2], angiogenesis is prolonged but still *self-limited*; however, this is not true of tumor angiogenesis which, once begun, continues indefinitely until the entire tumor is eradicated or the host dies. Without blood vessels, tumors cannot grow beyond a critical size (1–2 mm) or metastasize to another organ.

Angiogenesis is one of the most complex dynamic processes in biology, and is highly regulated by a balance of pro- and anti-angiogenic molecules. It is now widely accepted that the "angiogenic switch" is "off" when the effects of pro-angiogenic molecules is balanced by that of anti-angiogenic molecules, and "on" when the net balance is tipped in favor of angiogenesis [1,3]. Pro- and anti-angiogenic molecules can be secreted from cancer cells, endothelial cells, stromal cells, blood, and the extra-cellular matrix [4,5], the relative contributions of which are likely to change with tumor type and site, as well as with tumor growth, regression and relapse [1].

Although considerable advances have been made in our molecular and cellular knowledge of the *promotion*, *mediation *and *inhibition *of angiogenesis, very little is known about its underlying complex *dynamics*. Vasculature and more generally tubular organs develop in a wide variety of ways involving many cell processes [6-8].

In mathematical terms, angiogenesis is a *non-linear dynamic system *that is discontinuous in *space *and *time*, but advances through qualitatively different *states*. The word *state *defines the configuration pattern of the system at any given moment, and a dynamic system can be represented as a set of different states and a number of *transitions *from one state to another over a certain time interval [9,10].

**Figure 1:**
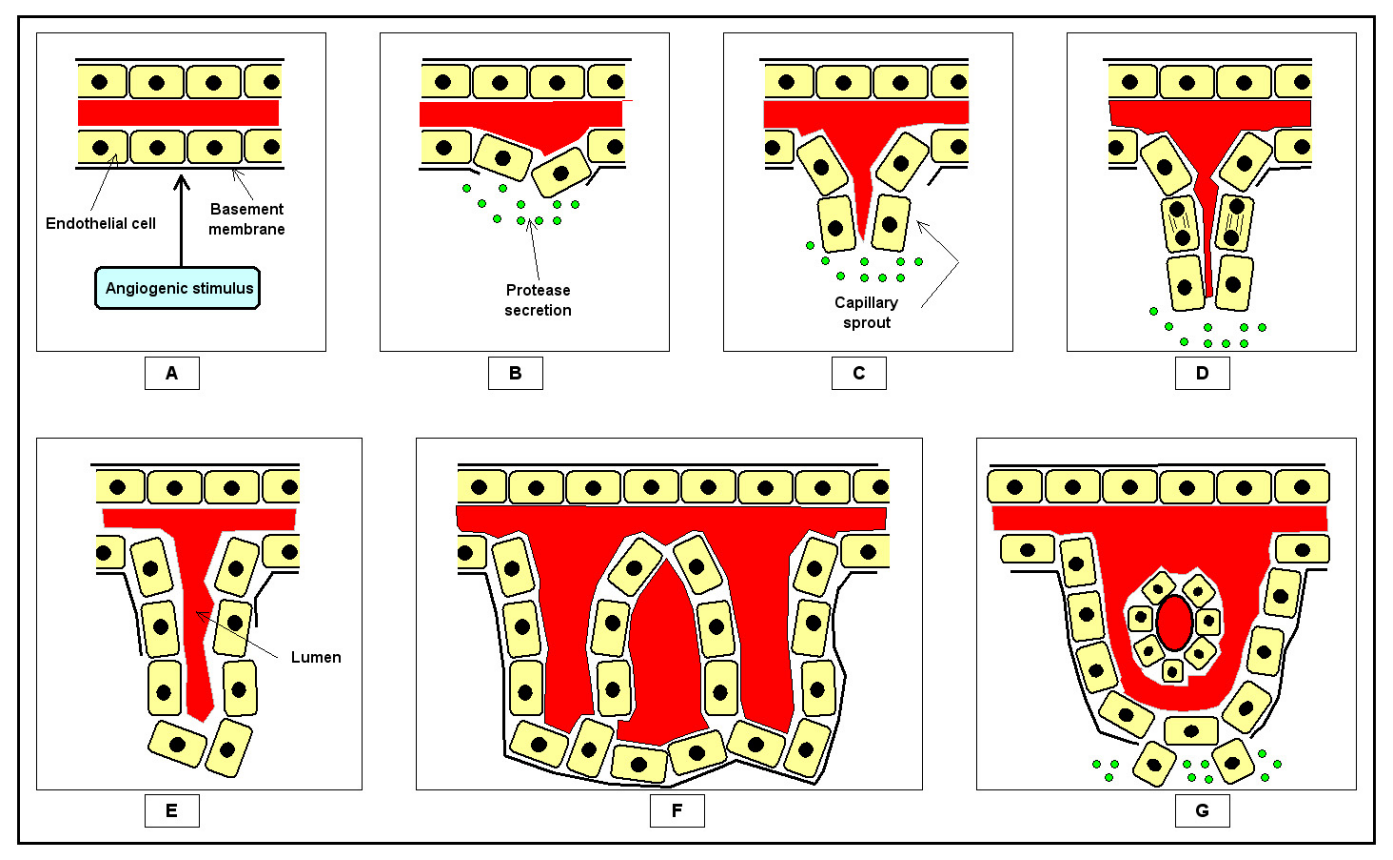
Angiogenesis is a complex dynamic process that evolves through different *states *and a number of *transitions *between two successive states. At least seven critical steps have so far been identified in the sequence of angiogenic events on the basis of sprout formation.

At least seven critical steps have so far been identified in the sequence of angiogenic events on the basis of sprout formation: *a) *endothelial cells are activated by an angiogenic stimulus; *b) *the endothelial cells secrete proteases to degrade the basement membrane and extra-cellular matrix; *c) *a capillary sprout is formed as a result of directed endothelial cell migration, *d) *grows by means of cell mitoses and migration, and *e) *forms a lumen and a new basement membrane; *f) *two sprouts come together to form a capillary loop; and *g) *second-generation capillary sprouts begin to form [1,11,12] (Fig. 1).

**Figure 2:**
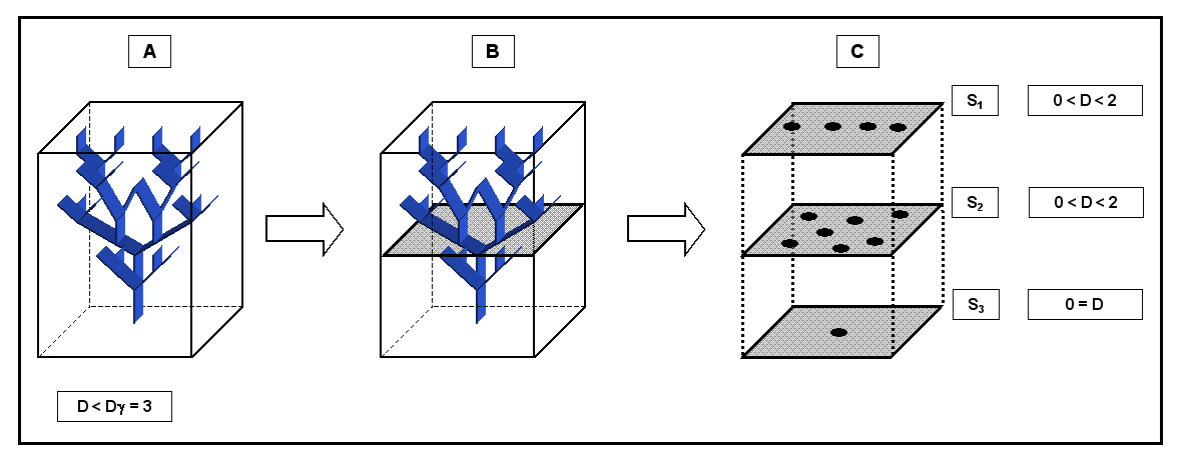
The space-filling property of the vascular system is quantified by the fractal dimension (D), which falls between two topological integer dimensions. **A**. A Euclidean three-dimensional space (*i.e. *a cube) can contain a branching structure (*i.e. *the vascular system) without this entirely filling its internal space. **B**. Two-dimensional sectioning of the vascular network makes it possible to identify a variable number of vessels depending on the *geometrical complexity *of the system at any particular level of sectioning. **C**. The *geometrical complexity *of a 2-D section (*s_1_*, *s_2_*, *s_3_*) of the vascular network depends in the number of sectioned vessels and their distribution pattern.

The progression of these states generates a complex ramified structure that irregularly fills the surrounding environment (Fig. 2). The main feature of the newly generated vasculature is the structural diversity of the vessel sizes, shapes and connecting patterns.

Tumor vessels are structurally and functionally abnormal [1,3]: unlike normal vessels, they are highly disorganized, tortuous and dilated, and have uneven diameters, and excessive branching and shunts. This may be mainly due to the heterogeneous distribution of angiogenic regulators, such as vascular-endothelial growth factor (VEGF), basic fibroblastic growth factor (bFGF) and angiopoietin [5,13], leading to chaotic tumor blood flow, and hypoxic and acidic tumoral regions [5,14-16]. Moreover, although it is commonly believed that the endothelial cells making-up tumor vessels are genetically stable, diploid cells (and thus different from genetically unstable neoplastic cells), tumor vasculature seems to be much more unpredictable [17].

These conditions all reduce the effectiveness of treatments, modulate the production of pro- and anti-angiogenic molecules, and select a subset of more aggressive cancer cells with higher metastatic potential [1].

A large number of clinical trials of anti-angiogenic therapies are being conducted throughout the world, but investigators are still concerned about how to achieve the maximum benefit from them and how to monitor patient response. There are currently no markers of the net angiogenic activity of a tumor that can help investigators to design specific anti-angiogenic treatment strategies [5,18], but it is reasonable to resume that the quantification of various aspects of tumor vasculature may provide an indication of angiogenic activity.

One often-quantified element of tumor vasculature is microvessel density (MVD), which is used to allow a histological assessment of tumor angiogenesis. The results of studies carried out over the last decade have suggested the value of using tumor MVD as a prognostic index in a wide variety of solid cancers, and it has also recently been assumed that MVD may reveal the degree of angiogenic activity in a tumor. On the basis of these assumptions, the quantification of MVD is thought to be a surrogate marker of the efficacy of anti-angiogenic agents as well as a means of assessing which patients are good candidates for anti-angiogenic therapy. However, MVD has a number of substantial limitations, mainly due to the complex biology characterizing tumor vasculature [17], and the highly irregular geometry that the vascular system assumes in *real space*, which cannot be measured using the principles of Euclidean geometry because it is only capable of interpreting regular and smooth objects that are almost impossible to find in Nature.

However, quantitative descriptors of its geometrical complexity can be usefully abstracted from the fractal geometry introduced by Benoit Mandelbrot in 1975 [20,21]. We here discuss the *surface fractal dimension (D*_*S*_*) *as a quantitative index of the 2-D *geometrical complexity *of vascular networks and their behavior during computer-simulated changes in *vessel density *and *distribution*.

## Geometrical properties of a vascular network

The human vascular system can be geometrically depicted as a complex fractal network of vessels that irregularly branch with a systematic reduction in their length and diameter [19].

Fractal objects are mainly characterized by four properties: a) the *irregularity *of their shape; b) the *self-similarity *of their structure; c) their non-integer or *fractal dimension*; and d) *scaling*, which means that the measured properties depends on the *scale *at which they are measured [22].

One particular feature of fractal objects is that the schemas defining them are continuously repeated at decreasing orders of magnitude, and so the form of their component parts is similar to that of the whole [20,21]: this property is called *self-similarity*. Unlike *geometrical self-similarity*, which only concerns mathematical fractal objects in which every smaller piece is an exact duplicate of the whole (e.g. Koch's snowflake curve, Sierpinski's triangle and Menger's sponge), *statistical self-similarity *concerns all complex anatomical systems, including tumor vasculature. The smaller pieces constituting anatomical entities are rarely identical copies of the whole, but more frequently "similar" to it and, in such systems, the *statistical properties *of the pieces are proportional to the statistical properties of the whole [23].

**Figure 3:**
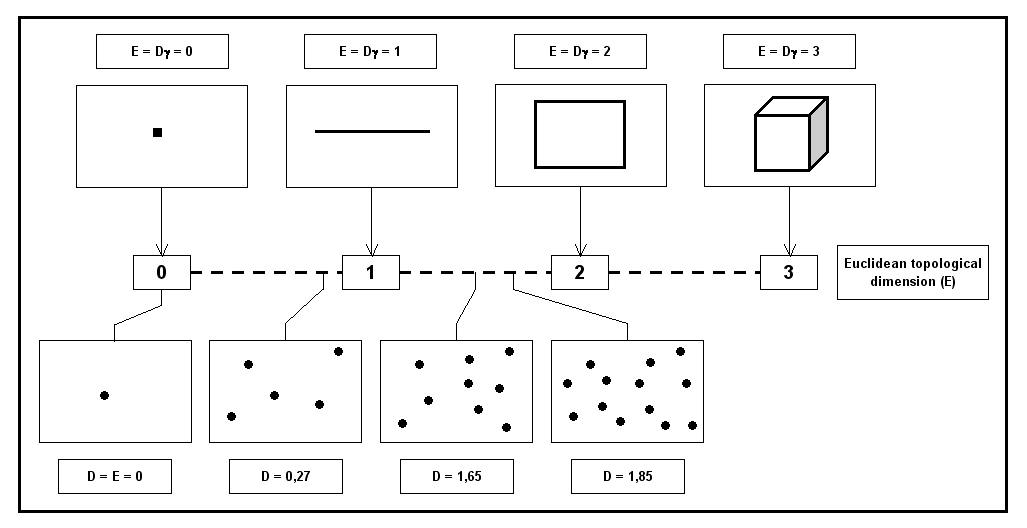
Fractal dimensioning of the 2-D *complexity *of a vascular network. The figure shows four idealized cross-sectioned vascular patterns that not only have a different number of vessels, but also clearly different distributions: the *geometrical complexity *arising from these two variables determines the value of the surface fractal dimension.

*Dimension *is a numerical attribute of an object that does not depend on its process of generation, and has been defined in two ways. The first is the *topological *or *Euclidean dimension *(Fig. 3), which assigns an integer to every point or set of points in *Euclidean space *(*E*): *0 *to a *point *(defined as that which has no part); *1 *to a *straight line *(defined as a length without thickness), *2 *to a *plane surface *(defined as having length and thickness, but no depth); and *3 *to *three-dimensional figures *(a volume defined by length, thickness and depth). The second was introduced by the mathematicians Felix Hausdorff and Abram S. Besicovitch, who attributed a *real number *to every natural object in *E *lying between the topological dimensions *0 *and *3 *(Fig. 3).

Benoit Mandelbrot uses the symbol *D*_*γ *_to indicates the topological dimension, and the symbol *D *to indicate that of Hausdorff-Besicovitch (also called the *fractal dimension*). The *D*_*γ *_and *D *of all Euclidean figures are *coincident *(*D*_*γ *_*= D*), but this is not true of fractal objects in which *D *is always >*D*_*γ*_.

**Figure 4:**
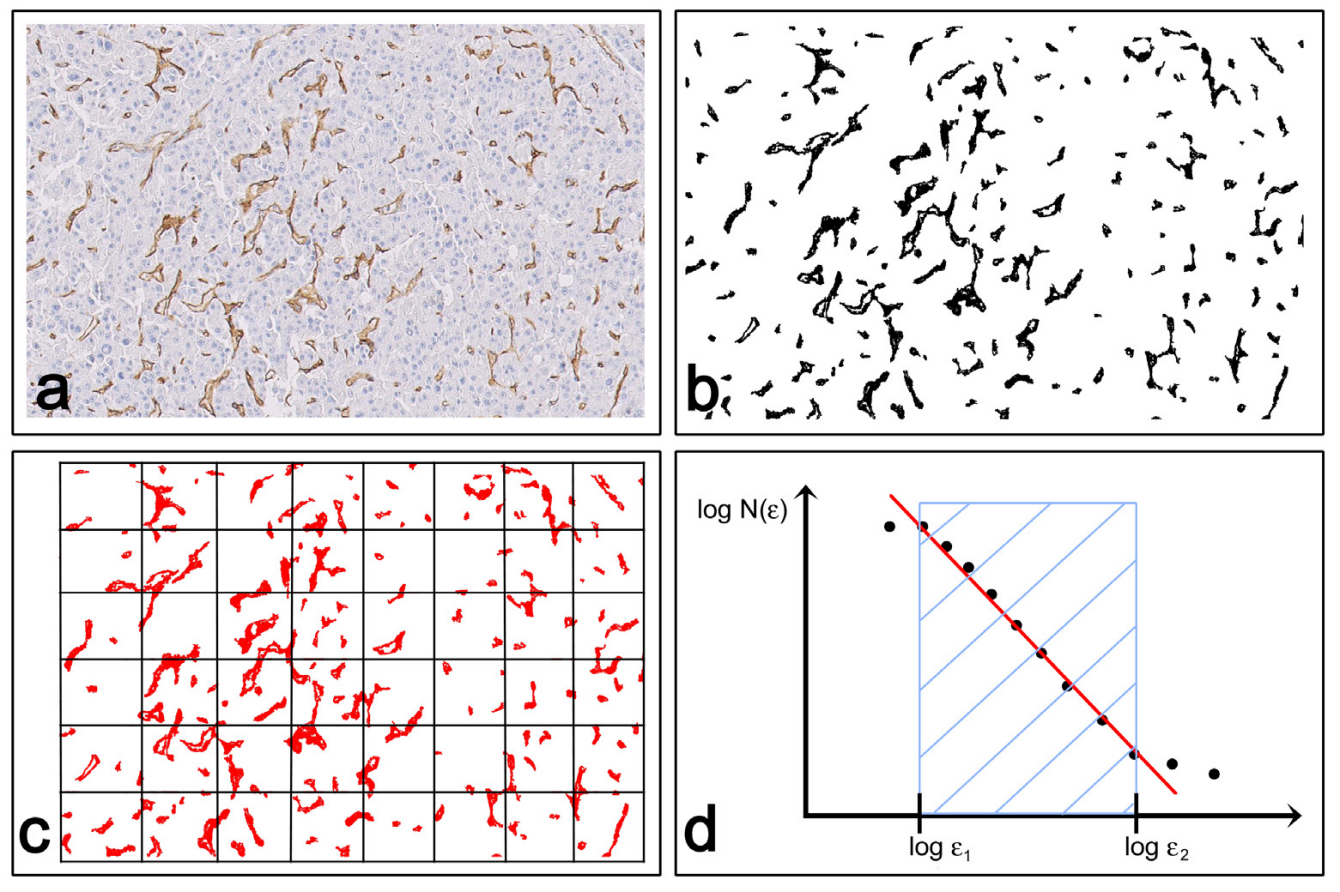
Computer-aided estimate of the surface fractal dimension *(D_s_) *of a vascular network in 2-D biopsy sections. **A**. Hepatocellular carcinoma section stained with antibodies raised against CD31 (Dako, Milan, Italy) that specifically react with vessels. **B**. Image *segmentation*: immunopositive vessels are specifically selected on the basis of the similarity of the color of adjacent pixels. **C**. Determination of *D*_*s *_using the box-counting algorithm. Briefly, the method counts the number of boxes of length *ε *required to cover the object being measured, indicated as N(*ε*). **D**. Prototypical curve obtainable using the box-counting method that highlights the so-called fractal windows ranged by box size *ε*_1 _and *ε*_2_, and represents the appropriate region in which to estimate the dimension. Box sizes of more than *ε*_2 _approach the size of the image until one box covers it completely, at which point N(*ε*) = 1 and the slope = 0. Box sizes smaller than *ε*_1 _approach a single pixel or the resolution of the image: in this region, box counting simply gives the area of the image.

As no anatomical entity corresponds to a regular Euclidean figure, their dimension is always expressed by a non-integer number falling between two integer topological dimensions. In our case (Fig. 2), the vascular network has a dimension lying between *2 *(plane surface) and *3 *(volume), and any two-dimensional section of a vascular system (as in the case of a histological section) has a dimension lying between *0 *(the dimension of a single isolated point) and *2 *when the sectioned vessels entirely fill a plane surface (Figs. 3 and 4).

Anatomical structures are also *hierarchical systems *that operate at different *spatial *and *temporal scales*, and different patterns can change, appear or disappear depending on the scale of magnification [22]. A fundamental characteristic is that the process operating at a given scale cannot be important at higher or lower scales [23].

The irregularity and self-similarity underlying *scale *changes are the main attributes of the architectural complexity of both normal and pathological biological entities [22-26]. In other words, the shape of a self-similar object does not change when the scale of measure changes because every part of it is similar to the original object; however, the *magnitude *and other *geometrical parameters *(e.g. the outline perimeter) of an irregular object differ when inspected at increasing resolutions that reveal an increasing number of details [25]. Over the last decade, accumulating experimental evidence has shown that the fractal patterns or self-similar structures of biological tissues can only be observed within the *scaling window *of an experimentally established measure of length *ε*_1_-*ε*_2 _(Fig. 4), within which experimental data sets follow a straight line with a slope *(1-D)*: i.e. the fractal dimension remains invariant at different magnifications [20-27].

## Methods

### Computer-aided modeling of two-dimensional vascular tree complexity

We have developed a computer model to simulate the geometrical complexity of a histological two-dimensional section of a tumor vascular tree that automatically generates an unlimited number of images with a changeable density of vessels irregularly distributed on a planar surface.

**Figure 5:**
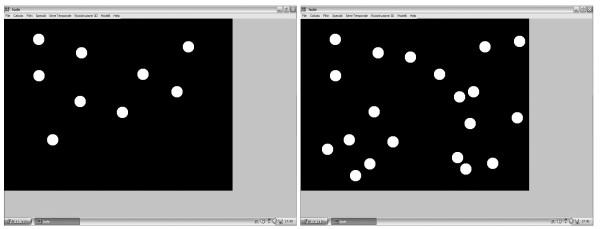
Computer-aided procedure used to quantify the surface fractal dimension of a simulated two-dimensional image of the vascular system. Prototypical 2-D simulated microscopy images of the vascular system with different vessel numbers and distribution were automatically generated, and their *D*_*s *_was determined.

In order to simplify the model, we considered all of the vessels as rounded, unconnected objects of equal magnitude (Fig. 5). As the parameters of a model must be as few as possible and it is necessary to reduce mathematical complexity [28-30], we included only two variables: *a) *the number of vessels; and *b) *their distribution in the surrounding environment. The vessel distribution patterns were randomly generated using different time-dependent seeds for random number function generation.

One thousand images were automatically generated for each vessel density (from five to 50 vessels, with the number being increased by five in each group), and their *D*_*S *_were estimated using the *box-counting method *[22].

*D*_*S *_was automatically estimated using the equation:


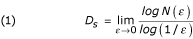


where *ε *is the side-length of the box, and *N*(*ε*) the smallest number of boxes of side *ε *required to completely contain the irregular object (Fig. 4).

As the zero limit cannot be applied to biological images, *D*_*S *_was estimated by means of the equation:

*(2) *    *D*_*S *_= *d*

where *d *is the slope of the graph of *log [N(ε)] *against *log (1/ε)*, in a fixed range of side-lengths (*ε *_1_-*ε *_2_) empirically evaluated by visualization [20-29].

### Statistical analysis

All of the data are expressed as mean values ± standard deviation, and the results were analysed using the Statistica software package (StatSoft Inc. Tulsa, USA). Unvaried analysis was performed by means of the Student *t *as required for parametric variables. *p *values of less than 0.05 were considered statistically significant.

## Results

**Figure 6:**
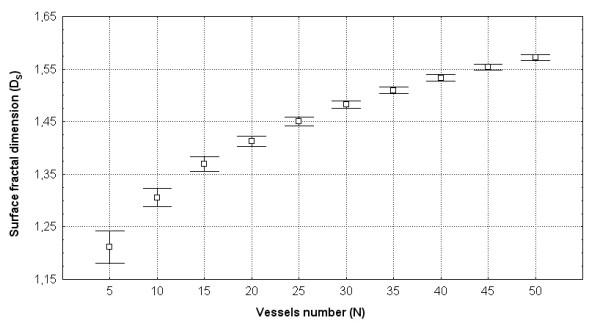
The behavior of *D*_*s *_during a simulated increase in vessel density. The graph shows that different *D*_*s*_values can be obtained for images with the same vessel density. As the only variable in these images is their distribution pattern, *D*_*s *_depends on the irregular arrangement of the vessels in the surrounding environment (note the standard deviation of each cell density group). *D*_*s *_also increases significantly when a higher vessel density is introduced into the system because of the greater space filled by the vascular component. The increase in vessel density reduces the variability of their space-filling properties, thus reducing the standard deviation.

The computer-aided simulations showed that different *D*_*S *_values can be obtained for images with the same vessel density (Fig. 6). As the only variable in these images is the vessel distribution pattern, *D*_*S *_depends on the irregular arrangement of the vessels in the surrounding environment. *D*_*S *_also significantly increased (*p *<0.05) when higher vessel densities were considered in the system (Fig. 6) because of the greater space filled by the vascular component (as shown in Fig. 3); the increased density of the vessels reduces the variability in their space-filling properties, and thus the standard deviation (Fig. 6).

## Discussion and conclusions

One of the most important and distinctive characteristics of biological systems is the *complexity *of their shape (*geometrical *or *spatial *complexity) and functions (*behavioral *complexity). Complexity is a real quality of organized biological matter that is mainly manifested in the living world as *diversity *and *organization*. No two anatomical systems are exactly alike because of the enormous variability not only between the different members of a population, but also between the component parts of an organism. The word complexity has long been used descriptively in order to describe, for example, a large number of genes or cellular interconnections [33], but complexity can also reside in the *structure *of a system (*i.e. *an intricate architecture or the existence of many different component parts with varying interactions) or its *non-linear functions *(*i.e. *physiological rhythms are rarely strictly periodic but fluctuate irregularly over time) [34].

The vascular system is a complex network consisting of branched tubes of different sizes that are irregularly settled in the surrounding environment [6,7]. This geometrical characteristic highlights the complexity of its generating process in *space *and *time*, and greatly biases any quantitative method that tends to idealize it as a smooth and regular Euclidean object.

However, both normal and tumor vasculature can more properly be considered *fractal objects *because of their irregular shape (*spatial conformation*), self-similar structure, non-integer dimension and dependence on the scale of observation (*scaling effect*) [19,35-37].

We here discuss the estimate of *D*_*S *_as a quantitative index of the 2-D spatial complexity of the vascular tree, in order to provide a closer-to-reality measure of this complex anatomical entity (Figs. 3 and 4).

The theory underlying *D*_*S *_was abstracted from fractal geometry, which is also called the *geometry of irregularity *[20,21]. The concept of spatial conformation has played a fundamental role in the study of biological macromolecules in chemistry (particularly biochemistry) since the early 1950s. However, it has only been introduced in the *science of morphology *as *theoretical morphology*, which studies extant *organismal forms *(complex structures of interdependent and subordinate elements whose relationships and properties are largely determined by their function in the whole) as a subset of the range of theoretically possible morphologies [32].

The significance of *D*_*S *_also comes from the fact that, like any other complex biological system, the vascular tree cannot be correctly quantified by measuring its individual properties (i.e. micro-vessel density, MVD). *D*_*S *_is a parameter that depends on: *a) *the number of vessels; *b) *the spatial relationships between the vascular components; and *c) *the interactions between the vascular components and the surrounding environment. In other words, its estimate is "ecologically" important because it provides a quantitative index of the "habitat structure".

As computer models are crucial for scientific procedures, and the modeling process itself represents the hypothetical-deductive approach in science [30-32], we developed a simple computer-aided model capable of generating an unlimited number of 2-D images of a simulated vascular network. The model was simplified by using a minimum amount of mathematical complexity and only two variables: the number of vessels and their pattern of distribution. A total of 10,000 images showing a different number of unconnected vessels irregularly distributed on a planar surface were automatically generated (Fig. 5) and, interestingly, it was found that *D*_*S *_increased with the number of vessels making up the system (Fig. 6); furthermore, its value changed when the same number of vessels were differently distributed in the surrounding environment.

In other words, it is plausible that an equal number of vessels may have different space-filling properties depending on their distribution pattern. These results suggest the usefulness of this model when comparing real vasculature configurations in order to explore the morphological variability that can be produced in nature, as it is now well known that aberrant vascular architectures in tumors may affect the uniform delivery of specific drugs to all cancer cells [15].

The model also suggests that:

*a) D*_*s *_can be an estimate of the 2-D geometrical complexity of the vascular system. As 2-D vascular complexity depends on the number of vessels and their distribution pattern, the use of MVD quantification alone to measure the angiogenic dependence of a tumor is strongly biased because the number of vessels does not reflect the number of tumor cells that can be supported by a vessel. Moreover the metabolic needs of cancer cells vary with the tissue of origin and change with tumor progression [18].

*b) D*_*S *_depends on the degree of vessel *contiguity *and *continuity*. These two geometrical properties determine what is called the *intercapillary distance*, and are not only involved in the spatial complexity of tumor vasculature, but also reflect the inviolable demand of a growing tumor for sufficient levels of nutrition and oxygen exchange. Inter-capillary distances are locally defined by the net balance between pro- and anti-angiogenic molecules in each microtissue region, as well as by non-angiogenic factors such as the oxygen and nutrient consumption rates of tumor cells. In normal tissue, vessel density fairly accurately reflects cell metabolic demands because evolutionary pressures have led to close and efficient coupling between vascular supply and metabolic needs. In tumors, the close coupling between vascular density and oxygen or nutrient consumption (*i.e. *the environment) may be loosened [18], thus altering not only the number of vessels but also the whole vascular architecture [15,38].

*c) D*_*S *_falls between 0 (corresponding to the Euclidean dimension of a point) and 2 (the dimension of a plane). The more *D*_*S *_tends towards 2, the more the analyzed vascular configuration tends to fill a 2-D space and the greater its geometrical complexity.

In conclusion, the present study indicates that the *complex geometry *of tumor vasculature and its well-known biological characteristics [18] mean that vascular network cannot be measured on the basis of MVD estimates alone. These findings also support the findings of various authors who have shown the uselessness of MVD as a predictor of anti-angiogenic treatment efficacy or for stratifying patients in therapeutic trials [14,39-41].

Scientific knowledge develops through the evolution of new concepts, and this process is usually driven by new methodologies that provide previously unavailable observation. The potential broad applicability of the proposed quantitative index makes it possible to explore the range of the morphological variability of vasculature that can be produced in nature, thus increasing its diagnostic importance in cancer research.

## Abbreviations

Ds, Surface fractal dimension; 2-D, two-dimensional; VEGF, Vascular-endothelial growth factor; bFGF, basic fibroblastic growth factor; MVD, microvessel density.

## Competing interests

The author(s) declare that they have no competing interests.

## Authors' contributions

FG conceived, coordinated and designed the study and drafted the manuscript; CR, PC, BF, EEF, EC, MCI participated in designing the study and drafting the manuscript. All of the authors have read and approved the final manuscript.

## Pre-publication history

The pre-publication history for this paper can be accessed here:
